# Neuraxial Anesthesia and Cancer Recurrence Following Prostatectomy: Thinking Outside the Box

**DOI:** 10.3390/pharmacy13050120

**Published:** 2025-09-01

**Authors:** Maria P. Ntalouka, Panagiotis J. Vlachostergios, Metaxia Bareka, Konstantinos Dimitropoulos, Anastasia Michou, Ioannis Zachos, Aikaterini Bouzia, Ecaterina Scarlatescu, Vassilios Tzortzis, Eleni M. Arnaoutoglou

**Affiliations:** 1 Department of Anesthesiology, Faculty of Medicine, School of Health Sciences, University of Thessaly, Larissa University Hospital, 41110 Larissa, Greece; 2 Department of Urology, Faculty of Medicine, School of Health Sciences, University of Thessaly, University Hospital of Larissa, 41110 Larissa, Greece; 3 Department of Medical Oncology, IASO Thessalias Hospital, 41110 Larissa, Greece; 4 Division of Hematology and Medical Oncology, Department of Medicine, Weill Cornell Medicine, New York, NY 10065, USA; 5 Department of Urology, Aberdeen Royal Infirmary, Aberdeen AB25 2ZN, UK; 6 Department of Anaesthesia and Intensive Care, University of Medicine and Pharmacy “Carol Davila” Fundeni Clinical Institute, 050474 Bucharest, Romania

**Keywords:** neuraxial anesthesia, radical prostatectomy, cancer recurrence, overall survival, regional anesthesia, perioperative care

## Abstract

Radical prostatectomy is the standard of care for the treatment of early, clinically localized prostate cancer (PC). In addition to known clinical prognosticators, perioperative conditions and the type of anesthesia may affect clinical outcomes through several mechanisms that favor a tumor-propagating state, including activation of the sympathetic system, increased opioid requirements, and inflammation. In this review, we provide an overview of the impact of the perioperative period on PC prognosis and patient outcomes. A non-systematic literature review was conducted to investigate the possible association between neuraxial anesthesia and outcomes after radical prostatectomy (RP) for prostate cancer. The following keywords were used: “cancer recurrence” OR “cancer prognosis” OR “metastasis” AND “neuraxial anesthesia” AND “prostate cancer”. Eligible studies were summarized in the form of a narrative review. In the era of limited use of ERAS protocols, the implementation of neuraxial anesthesia was found to reduce mortality after RP for primary prostate cancer when compared to general anesthesia. Although there was no significant association between anesthetic technique and radiological or biochemical-free survival, regional anesthesia may have an impact on short-term survival in patients with severe comorbidities, involving pulmonary complications and thrombosis. The effect of anesthetic technique on PC patient outcomes remains elusive, although preliminary retrospective evidence suggests a possible positive effect of neuraxial anesthesia on patient outcomes. As the perioperative period is considered a vulnerable timeframe for these patients, the role of the leadership dyad of surgeon and onco-anesthesiologist is crucial.

## 1. Introduction

Cancer remains the leading cause of mortality and disability, even though knowledge of cancer biology and new therapeutic interventions continues to increase [1,2]. Prostate cancer (PC) is the most common malignancy in males and the second leading cause of mortality [3]. Radical prostatectomy (RP) is the standard treatment for patients with localized cancer and has been shown to have a positive impact on mortality rates. However, despite the positive impact of RP on mortality, 1 out of 4 patients still suffer a local recurrence or distant metastasis following primary tumor resection [3]. Tumor stage, Gleason score, lymph node status, surgical margins, and preoperative level of serum prostate-specific antigen (PSA) are considered the most important prognostic indicators for disease recurrence [3].

Increasing evidence on conditions during the perioperative period in patients with PC suggest that this is a potentially tumorigenic state that promotes cancer progression and the development of metastases [4,5,6]. The ability of tumor cells to survive and colonize distant tissues perioperatively is unclear and is influenced by several variables and events with immunomodulatory effects. The pathophysiology of metastasis involves a combination of surgical stress, inflammation, perioperative immune regulation, pain, and angiogenesis [7].

Several studies, mostly retrospective, have investigated the possible relationship between anesthesia and oncological outcomes. Several hypotheses were described whereby neuraxial anesthesia, together with the administration of local anesthetics, may reduce the likelihood of tumor spread by (a) limiting the afferent signals sent from the surgical field to the central nervous system (CNS), (b) reducing efferent activation of the sympathetic nervous system, (c) reducing endogenous opioid release and perioperative opioid requirements, and (d) reducing inflammation through a direct anti-inflammatory effect of local anesthetic [5,6]. However, although regional anesthesia may influence long-term outcomes, recent clinical evidence suggests that anesthetic technique per se may have minimal impact on oncological outcomes [6]. In this review, we provide an overview of the impact of the perioperative period on PC prognosis and patient outcomes. We also discuss the relevant evidence on neuraxial anesthesia and cancer recurrence in PC patients and the crucial role of the onco-anesthesiologist, oncology pharmacist, and perioperative teamwork in optimizing postoperative outcomes.

## 2. Materials and Methods

A non-systematic literature review was conducted on PubMed in February 2025 using the following keywords: “cancer recurrence” OR “cancer prognosis” OR “metastasis” AND “neuraxial anesthesia” AND “prostate cancer”. No time limitations were set. Appropriateness for inclusion was determined by the authors in an attempt to include a wide and unbiased range of relevant studies. Non-English studies, letters to the editor, studies with unavailable full text, and retracted studies were excluded. Original research articles were preferred over review articles. References in narrative/systematic reviews and meta-analyses were additionally searched for relevance. Articles were selected for presentation and discussion to the judgment of the authors (MPNt and PJV) and according to relevance, merit, and up-to-date content. Disagreements were resolved through consensus between the aforementioned reviewers; if necessary, an additional senior reviewer (EMA) was consulted. Each study was described by the name of the primary author and year of publication. Two authors (MPNt and PJV) extracted the following data from each study: (i) type of study, (ii) type of anesthesia, (iii) sample size, and (iv) outcome with relevant hazard ratio, if available.

## 3. Results

### 3.1. Literature Search

**Figure 1:**
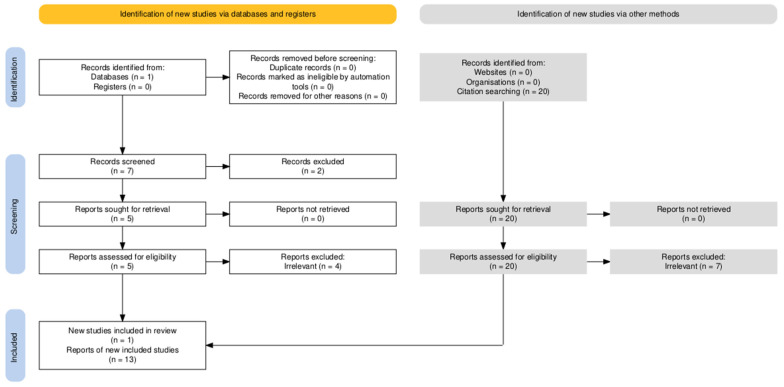
Prisma flowchart.

In total, seven articles were obtained through the database search. After application of inclusion and exclusion criteria, two records were excluded and five were assessed for eligibility. Four of these were deemed irrelevant, and finally one report was included. Additionally, 20 articled were found through citation searching and assessed for eligibility. Of them, 7 were deemed irrelevant, and finally, 13 articles were included. The final review therefore yielded a total of 14 studies (Figure 1).

### 3.2. The Impact of the Perioperative Period on Prostate Cancer Prognosis and Patient Outcomes

Even when surgery is performed with curative intent, the perioperative period appears to be a rather critical time during which cancer progression or distant metastases may occur through various pathophysiological mechanisms [1,5,6]. The tumorigenic nature of the perioperative period has been well established. Recent literature highlights that most surgical, anesthetic, and analgesic interventions may affect cancer cell metabolism and have long-term effects on disease recurrence and the ability to return to intended oncological treatment (RIOT) [8]. Therefore, the perioperative period has been referred to as the “perfect storm” for PC patients, as it can lead to dynamic cancer development and progression due to stress-induced immunosuppression and tumor cell proliferation. In addition, the surgical insult and certain drugs administered during the perioperative period can lead to epigenetic changes, altered gene transcription, and long-lasting effects on disease prognosis [9].

Several proposed mechanisms by which the perioperative period itself may have a direct and indirect impact on cancer cell survival include the surgical neuroendocrine and inflammatory response, subsequent relative immunosuppression, administration of certain pharmacological agents or transfusion of blood products, and the development of hypothermia and hypoxia [9,10]. Various factors can have a cancer-promoting or cancer-inhibiting effect, and it appears that when a patient’s immune balance is shifted towards immunosuppression, the cancer-promoting effects predominate and a suitable microenvironment for tumor growth is created [9,10]. Regarding the surgical neuroendocrine and pro-inflammatory stress response, surgical stress shows its “tumor-promoting effect” through activation of the hypothalamic–pituitary–adrenal axis and activation of the sympathetic nervous system, which contribute to the development of an immunosuppressive state. As a result of surgical stress, circulating levels of cortisol, catecholamines, proinflammatory cytokines, and prostaglandins are increased, natural killer cells and lymphocytes are impaired, antiangiogenic factors are decreased, and proangiogenic factors are increased. The elimination of tumor cells clearance is impaired, remaining cancer cells multiply, and the progression of the cancer is promoted [10].

In addition to the indirect effects on the tumor cells, the surgery itself also has a direct influence on the survival of the tumor cells [6]. The immunosuppressive state induced by the operation favors the growth of micro-metastases that were not diagnosed at the time of the operation and the formation of new metastases during primary tumor resection [4].

Surgical tumor manipulation is thought to be a risk factor for the release of cancer cells into the bloodstream and the spread of metastases to distant organs or intraperitoneal seeding and transcolonic spread. In addition, surgical incisions and tumor manipulation can lead to disruption of endothelia, changes in hydrostatic and oncotic pressure, and dissemination of tumor cells via lymphatics. Another cause of lymphatic or local cancer spread is minimal residual disease at the surgical margins. It is noteworthy that epithelial–mesenchymal transition (EMT) appears to be one of the most important and crucial steps in the metastatic process [6]. To successfully colonize a distant site and form a clinically detectable metastasis, circulating tumor cells (CTCs) must successfully undergo the following steps: (1) escape from the primary tumor, (2) intravasation, (3) circulation into the bloodstream, (4) extravasation by endothelial cells into the surrounding tissue, and (5) survival and proliferation in the tumor microenvironment through angiogenesis and immune escape [11]. EMT-phenotypic transformation of epithelial cancer cells into mesenchymal cancer cells allow mesenchymal cells to migrate, invade, and eventually resist apoptosis and colonize distant sites [6].

Enhanced recovery after surgery (ERAS) protocols, which include a combination of pre-, intra-, and post-operative measures and multimodal analgesia, have been proposed to minimize the aforementioned surgery-induced stress response and inflammation by optimizing the patient’s preoperative status and maintaining perioperative homeostasis [12]. Recently, Pang et al. [13] have shown that the implementation of ERAS protocols in oncological surgery can improve the timely initiation and completion of adjuvant post-operative chemotherapy [12,13]. In addition, high adherence, of up to 70%, to ERAS can lead to better outcomes. However, whether these short-term benefits of ERAS in patients undergoing cancer surgery can also lead to long-term benefits remains to be proven [12].

Although RP is a challenging surgical procedure, especially in elderly patients suffering from multiple comorbidities and malnutrition, there are still few guidelines for ERAS protocols for such major urological oncological procedures [12,13]. Table 1 lists the key ERAS elements and suggestions from the current literature that should be considered in patients undergoing RP to minimize the perioperative immunological stress response.

### 3.3. The Effect of Anesthetic Technique on Prostate Cancer

Over the past two decades, several studies have investigated the potential effects of neuraxial anesthesia/analgesia and local anesthetics on the outcomes of cancer treatment. Neuraxial techniques have been used in the perioperative care of patients with various primary cancers to control acute postoperative pain and reduce the consumption of opioids and volatile agent [2].

From a pathophysiological point of view, it seems quite tempting to expect an improvement in the prognosis of patients undergoing cancer surgery under neuraxial anesthesia [2]. It is known that neuraxial anesthesia, through its potent sympatholytic effect, can reduce the surgery-related stress response and has a dose-sparing effect on both opioids and volatile hypnotic agents which are associated with immunosuppressive effects and thus a poorer prognosis in cancer patients [4,5]. In addition, local anesthetics used in neuraxial anesthesia have direct anti-oncogenic and anti-inflammatory effects on tumor cells via different pathways. Local anesthetics activate apoptotic pathways, inhibit tumor cell growth and migration, increase natural killer cells activity and T-helper cells number, maintain interferon-gamma expression and signaling, increase interleukin-4 levels, and can reduce the production of interleukin-10, interleukin-8, and tumor necrosis factor alpha. Moreover, local anesthetics can also have direct anti-metastatic effects by inhibiting intracellular signaling events associated with angiogenesis, migration, and tumor invasion [1].

Several studies have investigated the effect of neuraxial anesthesia on tumor growth and the prognosis of PC patients (Table 2) [11,14,15,16,17,18,19,20,21,22,23,24,25,26]. However, studies comparing the effect of general anesthesia with neuraxial on the outcome of PC treatment did not provide conclusive or negative results [27,28]. A prominent systematic review of studies comparing general anesthesia and neuraxial anesthesia with or without general anesthesia was published in 2015 and included 10 studies with a total of almost 14.000 patients [28]. The vast majority of the studies were retrospective in nature, and one was a secondary analysis of a prospective randomized controlled trial (RCT) [28]. Only three studies reported positive results for neuraxial anesthesia, while the remaining seven studies were negative. The systematic review found no differences in overall survival, but this may be due to significant unmeasured confounding factors, as most studies were retrospective cohorts in which propensity matching was not applied. In the analysis that included only the studies that had performed propensity matching, the implementation of neuraxial anesthesia resulted in a statistically significant 19% reduction in mortality. This led to the conclusion that neuraxial anesthesia can reduce mortality after RP for primary prostate cancer. Regarding other endpoints, no significant association was found between the anesthesia technique and radiological progression-free survival (PFS) or biochemical-free survival. In addition, it has been suggested that regional anesthesia may have an impact on short-term survival in patients with serious comorbidities, involving pulmonary complications and thrombosis. However, these studies were conducted prior to the widespread implementation of ERAS protocols and showed no benefit in terms of PFS, biochemical-free survival, or RIOT [28].

Several limitations of the above studies make their interpretation difficult. Besides the retrospective nature of the vast majority of studies and the lack of randomization in seven of them, general anesthesia was performed in both patient groups. Moreover, the lack of a standardized anesthesia protocol for both the general and neuraxial anesthesia groups and the lack of a control group regarding the administration of anti-inflammatory or opioid medications pre- or post-operatively could represent additional confounding factors. Finally, the majority of patients included in these studies were characterized as being low-risk for cancer recurrence, which allowed the use of minimally invasive surgical techniques, thus limiting the generalizability of the conclusions regarding the type of anesthesia during RP in daily clinical practice [27,28,29].

### 3.4. Onco-Anesthesia, the Role of Perioperative Teamwork and Future Perspectives

In light of new findings about the potential impact of anesthesia techniques on the survival of cancer patients, a new anesthesia subspecialty, called onco-anesthesia, has been developed. Its main goal is to educated physicians on how perioperative interventions during oncologic surgery (including ERAS protocols and multimodal analgesia) can change the outcome by increasing the disease-free survival of cancer patients. In addition, the implementation of high-quality onco-anesthesia care aims to minimize perioperative morbidity and post-operative persistent cancer-related pain and develop an optimum plan for RIOT, with the ultimate goal of reducing cancer metastasis and recurrence [30].

The importance of multidisciplinary care and cooperation, especially the relationship between the anesthesiologist and the surgeon in urological–oncological operations, cannot be overestimated. A trusted team is essential for critical and major procedures in frail patients, and the principle of “the right anesthesiologist for the surgeon” is central to providing optimal, comprehensive patient-centered care that can lead to better outcomes [31]. In other words, optimal relationships and effective teamwork between surgeons and anesthesiologists in the implementation of ERAS protocols are key to the success of urologic surgery, including RP [31].

There are two main axes along which teamwork between the members of the perioperative team must ensure high-quality perioperative care. The first axis concerns patient safety and the second is related to optimal postoperative outcomes [31,32,33,34,35]. Despite the variability of perioperative teams consisting of multiple members, it is the relationship of the leadership dyad consisting of the anesthesiologist and the surgeon that should be held accountable for the overall performance of the team [31]. Therefore, the leadership dyad of surgeon and anesthesiologist during the perioperative period has been identified as the main determinant of the success or failure of the entire team [31,32].

With regard to the multidisciplinary perioperative care of PC patients, the important role of oncology pharmacists should also be emphasized. Oncology pharmacists have been involved in the care of cancer patients for over 50 years due to their in-depth clinical knowledge and understanding of cancer treatment [36]. However, their role is expanding, and they are actively involved in several cancer-related clinical pathways [36]. Regarding the perioperative period, it appears that both ambulatory and inpatient oncology pharmacists should play a central role in the ERAS pathway of cancer patients [36]. The ambulatory oncology pharmacist should be responsible for the pre- and post-discharge care of the cancer patient in terms of supportive care medication, anticancer medication adjustments, perioperative optimization, and patient education [36]. The inpatient oncology pharmacist should be responsible for the pharmacotherapy management of patients while they are hospitalized. They should work closely with the onco-anesthesia team in an attempt to further optimize the perioperative care of cancer patients in terms of safety and individualization. As oncology pharmacists play a central role in extending and improving patient care and their value to patient outcomes has long been demonstrated, it seems of paramount importance to include them in the multidisciplinary perioperative team of prostate cancer patients [36].

Consensus on the best anesthetic practices for patients undergoing cancer surgery remains challenging. Therefore, it is important to raise awareness among all specialties involved in the perioperative multidisciplinary care of operable cancer patients to optimize collaboration and research in this area. Obtaining high-quality data from well-designed prospective studies could support ongoing efforts to optimize the management of preoperative comorbidities, as well as the RIOT interval. In addition, cancer-related quality of life, days alive and out of the hospital, time to tumor progression, disease-free survival, cancer-specific survival, and overall survival should be included as endpoints in ongoing and future clinical trials. Investigating whether interventions such as prehabilitation in the form of exercise training and nutritional and psychological support can influence RIOT are important clinical questions that need to be addressed [9,30,35,37].

## 4. Conclusions

The influence of anesthetic technique on outcomes in PC patients remains elusive, although preliminary retrospective evidence suggests a possible positive effect of neuraxial anesthesia on patient outcomes. As the perioperative period is considered particularly vulnerable for cancer patients, the role of the leadership dyad of surgeon and onco-anesthesiologist is critical. A multidisciplinary patient-centered comprehensive approach aiming at optimal control of surgical stress response, inflammation, pain, and perioperative immunologic modulation is essential to prevent cancer progression and ensure the best possible care and prognosis for PC patients undergoing RP.

## Figures and Tables

**Table 1:** ERAS elements in major urologic surgery, including radical prostatectomy [12,13].

Preoperative period	Preadmission counselling and education Preoperative optimization Nutritional therapy Bowel preparation Thromboembolic prophylaxis Preoperative fasting Preoperative carbohydrate loading Antimicrobial prophylaxis Prevention of nausea and vomiting
Intraoperative period	Pre-emptive multimodal analgesia Goal-directed fluid therapy Preventing hypothermia Minimally invasive surgery
Postoperative period	Multimodal analgesia Optimal nasogastric tube duration Optimal urinary drainage Optimal drainage Prevention of postoperative ileus Early oral intake and postoperative nutrition Early mobilization

**Table 2:** Summary of studies in prostate cancer investigating the effect of neuraxial anesthesia.

Study [Author, Year]	Method	Intervention	RA Group [n = ]	Non-RA Group [n = ]	Notes
Biki, 2008 [11]	Retrospective	GA-EA vs. GA	103	102	EA showed a marginal benefit on RFS Hazard ratio 0.43 (0.22–0.83; *p* = 0.012)
Wuethrich, 2010 [14]	Retrospective	GA-EA vs. GA	103	158	EA showed a benefit on PFS [hazard ratio 0.45 (0.27–0.75; *p* = 0.002)] but no OS
Scavonetto, 2014 [15]	Retrospective, focused on PFS	GA-EA vs. GA	1642	1642	EA showed marginal benefit on OS [hazard ratio 1.32 (1.00–1.74; *p* = 0.047)] and improvement of RFS [hazard ratio 2.81 (1.31–6.05; *p* = 0.008)]
Tsui, 2010 [16]	RCT, sub-analysis	GA-EA vs. GA	40	50	EA showed no benefit on RFS Hazard ratio 1.33 (0.64–2.77; *p* = 0.44)
Forget, 2011 [17]	Retrospective	GA-EA vs. GA	578	533	Intravenous sulfentantil showed significantly improved RFS when compared with EA Hazard ratio 7.78 (5.79–9.78; *p* < 0.05)
Wuethrich, 2013 [18]	Retrospective	GA-EA vs. GA	67	81	EA showed no benefit on RFS [hazard ratio 0.91 (0.62–1.33; *p* = 0.6)] and OS [hazard ratio 1.79 (0.95–3.39; *p* = 0.07)]
Roiss, 2014 [19]	Retrospective	GA-Spinal vs. GA	3047	1725	Spinal showed no benefit on RFS [hazard ratio 0.82 (0.77–0.86; *p* = 0.65)], DFS [hazard ratio 0.96 (0.94–0.97; *p* = 0.11)], and OS [hazard ratio 0.95 (0.91–0.97; *p* = 0.41)],
Tseng, 2014 [20]	Retrospective	GA-EA vs. GA	1166	798	EA showed no benefit on RFS Hazard ratio 1.10 (0.85–1.42; *p* = 0.458)
Sprung, 2014 [21]	Retrospective, focused on PFS	GA-EA vs. GA	486	483	EA showed no benefit on cancer recurrence Hazard ratio 0.79 (0.60–1.04; *p* < 0.05)
Ehdaie, 2014 [22]	Retrospective	GA vs. Spinal	264	665	No benefit on RFS
Pikramenos, 2022 [23]	Prospective	CESA vs. GA	30	30	No benefit on 12-month oncological outcome
Tikuisis, 2009 [24]	RCT	GA-EA vs. GA	27	27	Reduced intraoperative blood loss (740 ± 210 mL versus 1150 ± 290 mL, *p* < 0.001) and allogenic transfusions 0.19 blood units transfused versus 0.52, *p* = 0.007) in GA-EA group
Frank, 1998 [25]	Retrospective	GA vs. EA vs. CESA	EA = 17 CESA = 143	32	EA showed benefit only on blood loss (*p* < 0.001) and hospital stay (*p* = 0.04)
Reeves, 1999 [26]	Retrospective, Matched-Cohort	GA vs. Spinal	70	68	No benefit to the overall outcome

GA: general anesthesia, EA: epidural anesthesia or analgesia, CESA: combined epidural spinal anesthesia, OS: overall survival, PFS: progression-free survival, RFS: recurrence-free survival, DFS: disease-free survival, RCT: randomized controlled trial.

## Data Availability

No new data were created or analyzed in this study. Data sharing is not applicable to this article.

## Abbreviations

The following abbreviations are used in this manuscript:

RP	Radical Prostatectomy
PC	Prostate Cancer
CNS	Central Nervous System
RIOT	Return to the Intended Oncologic Treatment
EMT	Epithelial-Mesenchymal Transition
CTCs	Circulating Tumor Cells
ERAS	Enhanced Recovery After Surgery
RCT	Randomized Controlled Trial
PFS	Progression-Free Survival
GA	General Anesthesia
EA	Epidural Anesthesia or Analgesia
CESA	Combined Epidural Spinal Anesthesia
OS	Overall Survival
RFS	Recurrence-Free Survival
DFS	Disease-Free Survival
